# Polymorphisms in circadian rhythm genes and the risk of differentiated thyroid cancer

**DOI:** 10.3389/fgene.2025.1539090

**Published:** 2025-06-04

**Authors:** Takiy-Eddine Berrandou, Emilie Cordina-Duverger, Claire Mulot, Anne-Valérie Guizard, Claire Schvartz, Pierre Laurent-Puig, Monia Zidane, Florent De Vathaire, Pascal Guénel, Thérèse Truong

**Affiliations:** ^1^ University Paris-Saclay, UVSQ, Inserm, Gustave Roussy, CESP, Team “Exposome and Heredity”, Villejuif, France; ^2^ Quantitative Genetics and Genomics (QGG), Aarhus University, Aarhus, Denmark; ^3^ Centre de ressources Biologiques EPIGENETEC, Centre de Recherche des Cordeliers, Université Paris Cité, Sorbonne Université, INSERM, Paris, France; ^4^ Registre Général des tumeurs du Calvados, Centre François Baclesse, Caen, France; ^5^ Inserm U1086 -UCN “ANTICIPE”, Caen, France; ^6^ Registre des Cancers Thyroïdiens, Institut GODINOT, Reims, France; ^7^ University Paris-Saclay, UVSQ, Inserm, Gustave Roussy, CESP, Team “Epidemiology of radiations”, Villejuif, France

**Keywords:** differentiated thyroid cancer, case-control study, circadian rhythm, gene–environment interaction, pathway analysis

## Abstract

**Introduction:**

Circadian rhythms are controlled by biological clocks regulated at the molecular level by a set of circadian genes operating through a negative feedback loop. These genes also regulate key biological processes, including cell proliferation, cell cycle, and apoptosis.

**Methods:**

We investigated the role of circadian gene polymorphisms in the risk of differentiated thyroid cancer (DTC) and their interaction with DTC risk factors. Data were obtained from 463 DTC cases and 482 unrelated controls of European ancestry, selected from two population-based case-control studies conducted in France. Associations with 570 single nucleotide polymorphisms (SNPs) in 23 circadian genes were evaluated using multivariate logistic regression models. Gene- and pathway-level associations and gene–environment interactions were analyzed using the adaptive rank truncated product (ARTP) method.

**Results and discussion:**

We found no significant association between DTC risk and circadian gene polymorphisms at the SNP, gene, or pathway levels. However, we observed statistically significant interactions between smoking status and SNPs rs11204897 (*RORC*) and rs1012477 (*PER3*), as well as with the *PER3* gene and the overall circadian pathway. These results suggest that smoking status may modulate the association between DTC and polymorphisms in circadian genes. Further studies are needed to confirm these findings.

## Introduction

Thyroid cancer is the most common malignancy of the human endocrine system. Its incidence is approximately three times higher in women than in men. It is also characterized by wide geographic variations around the world and by a steady increase over the past four decades in Western countries (Colonna et al., 2010). This increase in incidence has been linked to enhanced detection of micro-cancers as a result of changes in medical screening practices (Leenhardt et al., 2004; La Vecchia et al., 2015), but it has also been linked to changes in lifestyle factors and environmental exposures (Pellegriti et al., 2013). Papillary thyroid carcinoma (PTC) and follicular thyroid carcinoma (FTC) are the most common histologic subtypes of differentiated thyroid carcinomas (DTCs), and they account for approximately 90% of all thyroid cancers. Exposure to ionizing radiation during childhood (Iglesias et al., 2017) and overweight (Ma et al., 2015) have been consistently associated with DTC. Deficiency or excess of iodine intake, high parity, and later age at menarche have also been suspected to increase the risk (Cordina-Duverger et al., 2017; Liu et al., 2017), whereas oral contraceptive use, alcohol consumption, and tobacco smoking have been reported to decrease the risk of DTC (Truong et al., 2005; Leux and Guenel, 2010; Kabat et al., 2012; Kitahara et al., 2012). Thyroid cancer is one of the cancers with the highest familial risk (Hemminki and Vaittinen, 1999), with relative risk estimates for a history of thyroid cancer in first-degree relatives ranging from 3 to 8 (Frich et al., 2001; Mack et al., 2002; Hemminki et al., 2005), suggesting an important role for genetic risk factors. Although the associations between DTC risk and genomic loci reported in linkage analysis studies have not been consistently replicated (Malchoff et al., 2000; McKay et al., 2001; Cavaco et al., 2008; He et al., 2009; Suh et al., 2009), a few susceptibility loci were identified in genome-wide association studies (GWAS), but they showed only modest effects (Gudmundsson et al., 2009; Takahashi et al., 2010; Gudmundsson et al., 2012; Figlioli et al., 2014; Mancikova et al., 2015; Truong et al., 2021), suggesting that additional loci remain to be discovered.

In parallel, significant progress has been made in understanding the somatic alterations involved in thyroid carcinogenesis. DTCs are characterized by mutually exclusive driver events, primarily involving the *MAPK* and *PI3K/AKT* signaling pathways (Truong and Lesueur, 2022; Nannini et al., 2023). In PTC, the most common mutations affect *BRAF*—particularly the V600E variant, which is found in approximately 60% of cases—followed by *NRAS* (4%), HRAS (1.5%), and *KRAS* (0.3%), whereas FTC more frequently harbors *RAS* mutations (∼40%) or *PAX8/PPARG* rearrangements (Truong and Lesueur, 2022; Nannini et al., 2023). Rearrangements involving *RET* (8%), *BRAF* (2%), *NTRK1/3* (2%), and *ALK* (1%) are also observed, often in association with prior radiation exposure (Truong and Lesueur, 2022; Nannini et al., 2023). Moreover, promoter mutations in the *TERT* gene are found in approximately 12% of PTC and 18% of FTC, and their co-occurrence with *BRAF* V600E mutations has been consistently associated with poorer prognosis and increased risk of recurrence (Mady et al., 2020; Nannini et al., 2023). These somatic alterations not only serve as diagnostic and prognostic markers but also inform personalized therapeutic strategies—for instance, the clinical benefit of *RET* and *NTRK* inhibitors in patients with advanced, radioactive iodine-refractory DTC (Truong and Lesueur, 2022; Nannini et al., 2023).

Circadian genes are among the genes that might be involved in thyroid carcinogenesis. These genes control most physiological body functions that operate on a circadian (i.e., approximately 24 h) rhythm, such as the sleep/wake cycle, body temperature, or hormone secretion (Ikegami et al., 2019). The circadian rhythm is regulated at the molecular level by the circadian genes, which are organized in a complex network that functions through multiple periodic transcription–translation feedback loops (Ikegami et al., 2019). Circadian genes are involved in oncogenic mechanisms and may increase cancer susceptibility (Fu et al., 2002; Gery et al., 2006; Hua et al., 2006; Lee, 2006; Gery et al., 2007; Sahar and Sassone-Corsi, 2007; Hoffman et al., 2008; Xiang et al., 2008). Night shift work, which involves the disruption of the circadian rhythms caused by misalignment of the sleep/wake cycle with the natural light/dark cycle, was classified as a probable carcinogen by IARC in 2019 (IARC Working Group on the Identification of Carcinogenic Hazards to Humans, 2020).

Although circadian gene polymorphisms have been associated with the risk of breast and prostate cancers (Zhu et al., 2009; Karantanos et al., 2014; Truong et al., 2014; Valenzuela et al., 2016; Benna et al., 2017; Gu et al., 2017; Mocellin et al., 2018; Wendeu-Foyet et al., 2019), to the best of our knowledge, no study has investigated the role of circadian gene polymorphisms in constitutional DNA. Nevertheless, experimental studies on thyroid tumor tissues have shown that the expression of some circadian genes (*ARNTL*, *CRY*, *PER*, *RORC*, *NR1D1*, and *BTRC*) was deregulated or repressed compared to that in healthy tissues (Shaik et al., 2012; Fu and Kettner, 2013; Mannic et al., 2013; Mond et al., 2014), possibly indicating a role of these genes in thyroid carcinogenesis.

In this paper, we tested the hypothesis that circadian gene polymorphisms are associated with the risk of DTC. Given that the circadian gene pathway is a complex network involving multiple interactions between genes, we used a gene-set approach to examine the joint genetic effects of these variants at the gene and circadian pathway levels. In addition, we also explored the interactions of circadian gene variants with suspected lifestyle risk factors of DTC at the SNP, gene, and pathway levels.

## Materials and methods

### Study population

Data from two population-based case-control studies were used in the analysis. Study protocols were approved by the local ethics committees, and written informed consent was obtained from all participants.

### CATHY study

The CATHY study was described in detail previously (Cordina-Duverger et al., 2017). In brief, we conducted a population-based case-control study in three French departments covered by a cancer registry: Calvados, Marne, and Ardennes. Cases involved DTC patients aged 25 years and older who were diagnosed between 1 January 2002 and 31 December 2007. Controls were selected at random from the telephone directory and frequency-matched to the cases by sex, age (5-year age groups), and study area. To prevent possible selection bias arising from differential participation rates across categories of socioeconomic status (SES) among controls, quotas by SES were applied to reflect the distribution by SES categories in the general population of the department of residence. A total of 621 cases and 706 controls were recruited for the study.

### YOUNG-THYR study

The YOUNG-THYR study was described in detail previously (Xhaard et al., 2015). All patients who were diagnosed with DTC between 1 January 2002 and 31 December 2006, who were born after 1 January 1971, and whose primary residence was in one of the regions of eastern France (Alsace, Champagne-Ardenne, Corsica, Franche-Comté, Lorraine, Rhône-Alpes, or Provence-Alpes-Côte d'Azur) were eligible for this study. Cases were identified by three main sources, depending on the region: 1) the General Cancer Registry in Champagne-Ardenne, Alsace, and Rhône-Alpes; 2) the National Early Childhood Cancer Registry (which contains information on children <15 years of age) in all regions; and 3) private and public hospitals in Lorraine, Franche-Comté, Corsica, and Provence-Alpes-Côte d'Azur. In the latter four regions, which are not covered by the General Cancer Registry, the identification of cancer cases was performed in collaboration with local public health structures. Controls were selected from the general population by random selection from telephone directories and individually matched to cases on sex, date of birth (plus or minus 1 year), and region of residence. The study included 805 cases and 876 controls.

### Data collection

Cases and controls were interviewed in their homes by trained interviewers using standardized questionnaires. We collected information on sociodemographic characteristics, personal and family medical history, reproductive factors, anthropometric measurements, physical activity, tobacco smoking, alcohol consumption, recreational activities, and occupational and residential histories. At the end of the interview, participants were asked to provide a saliva sample (Oragene^®^) for DNA banking. DNA was genotyped in 810 women from the CATHY study who agreed to provide saliva (403 cases and 407 controls) and in 200 women randomly selected from the YOUNG-THYR study (100 cases and 100 controls).

### Genotyping

A dedicated genotyping chip was designed by the team to explore associations between genetic variants in candidate biological pathways relevant to the etiology of hormone-dependent cancers (steroid hormone metabolism, DNA repair genes, obesity genes, circadian genes, etc.) (Truong et al., 2014). In total, 28 pathways, including 652 genes, were selected in January 2011 from the Kyoto Encyclopedia of Genes and Genomes (KEGG) database and a literature review. A total of 8,716 SNP tags selected around each gene and capturing most of the genetic variation in these regions were selected by pairwise approach with r^2^ ≥0.8 with a minimum allele frequency (MAF) of 0.05 in the Caucasian population (CEU) genotyped using the HapMap project (21/phase II, NCBI build 36.1, assembly dbSNPb126). This chip has a high coverage of genes and pathways of interest compared to chips used in genome-wide association studies (GWAS). The chip was designed by Illumina, and genotyping was carried out by IntegraGen (Evry, France).

### Quality control

Of the 8,716 SNPs selected for inclusion in the chip, 987 could not be genotyped and 728 were excluded due to a genotyping rate (call rate) below 95%. Of the remaining 7,021 SNPs, one failed the Hardy–Weinberg equilibrium (HWE) test at p < 10^−5^ (Bonferroni correction based on 7,021 tests). Ten CEPH control subjects were also genotyped using the chip, and when more than one mismatch was observed for a given SNP with HapMap, the variant was excluded (n = 16). We also excluded 143 SNPs with MAFs less than 0.01 in controls. After all exclusions, 6,861 SNPs in 652 genes remained. In this study, we used the genotypic data obtained in the circadian rhythm pathway that includes 570 SNPs distributed in the following 23 genes: clock circadian regulator (*CLOCK*), *ARNTL*, neuronal PAS domain protein 2 (*NPAS2*), *CRY1*, *CRY2*, *PER1*, *PER2*, *PER3*, *RORA*, *RORB*, *RORC*, basic helix-loop-helix family member e40 (*BHLHE40*), *BHLHE41*, S-phase kinase associated protein 1 (*SKP1*), F-box and WD repeat domain containing 11 (*FBXW11*), cullin 1 (*CUL1*), timeless circadian regulator (*TIMELESS*), F-box and leucine rich repeat protein 3 (*FBXL3*), *NR1D1*, casein kinase 1 delta (*CSNK1D*), *CSNK1E*, ring-box 1 (*RBX1*), and *BTRC*. All SNPs included in the final analysis were in HWE with a threshold of p < 10^−5^. HWE was tested using the “genetics” package in R software (version 4.0.3).

Of the 1,010 DNA samples available (810 from CATHY and 200 from YOUNG-THYR), we excluded 32 women who could not be genotyped (25 from CATHY and seven from YOUNG-THYR), 1 woman from YOUNG-THYR who withdrew her consent, and 32 women identified as outliers in a principal component analysis (PCA) (19 from CATHY and 13 from YOUNG-THYR), leaving 945 women (463 cases and 482 controls) available for analysis. Of these, 766 were participants of the CATHY study (377 cases 389 controls) and 179 were participants of the YOUNG-THYR study (86 cases and 93 controls). We assessed relatedness between individuals using 8,716 SNPs and did not identify any related pairs. Self-reported ancestry was not available for 20% of participants. Among the 32 women excluded based on PCA, 85% of them had declared a non-European ancestry, supporting the use of PCA as an effective strategy to identify population structure and ensure genetic homogeneity in the final sample.

### Statistical analyses

#### SNP analysis

Odds ratios (ORs) of the association between DTC and SNPs in circadian genes were estimated by unconditional logistic regression assuming a log-additive genetic model. Each SNP was coded 0, 1, or 2, depending on the number of rare alleles in the genotype. We tested the interaction between genetic polymorphisms and DTC risk factors, including body mass index (BMI) 1 year before diagnosis (kg/m^2^, continuous), smoking status (never, former that quit smoking for at least 1 year, or current smoker), age at menarche (≤12, 13, and 14 or ≥15 years of age), parity (0/1/2/3/≥4 full term pregnancies), and oral contraceptive use (ever/never). Interaction with each of the 570 SNPs included in the circadian genes was tested using the likelihood ratio test comparing the models with and without an interaction term.

#### Gene and pathway analysis

We investigated the risk of DTC associated with each circadian gene (viewed as a set of SNPs) and with the circadian gene pathway (viewed as a set of 23 circadian genes) using the adaptive rank truncated product (ARTP) method (Yu et al., 2009). To calculate the p-value associated with a given gene, this method combines the p-values calculated for all SNPs within the gene. To calculate the p-value associated with the whole pathway, it combines the p-values calculated for all the genes within the pathway. The p-values at the gene (or pathway) level are calculated from the product of the “K” smallest p-values of SNPs within a gene (or the “K” smallest p-values of genes within a pathway, respectively). Different “K” (or “K”) truncation points were used depending on the number of SNPs in the gene (or the number of genes in the pathway, respectively). We chose the truncation points proposed by Yu et al. (2009), i.e., five truncation points defined at every top 5% SNPs in terms of p-value for the association at the gene level and ten truncation points defined at every top 5% genes in terms of p-value for the association between the pathway and thyroid cancer risk. The smallest product p-value over the different truncation points was chosen as the test statistic, and its significance was assessed using an empirical product distribution from data obtained by a permutation procedure of case-control status (n = 20,000) in the original database.

To investigate the interaction between DTC risk factors (BMI, smoking status, age at menarche, parity, and oral contraceptive use) and genetic factors at the gene and circadian pathway levels, the ARTP method was slightly modified (PIGE package in R) to use the “SNP x environment” interaction p-values as input data instead of the p-value of each SNP. In addition, instead of only permuting case-control status in the permutation phase, we permuted the case-control status with the epidemiological variables so that all epidemiological data from one individual are randomly assigned to another individual. This keeps the adjustment for known or suspected risk factors for thyroid cancers. In this way, linkage disequilibrium between SNPs is also preserved (as in the classical ARTP method).

All analyses were adjusted for the matching variables: age and department of residence, as well as for possible confounders: years of education (≤5, 6–9, 10–12, and >12), marital status (single; married or partnered; divorced or separated; and widowed), age at menarche (≤12, 13, 14, or 15 years and older), parity (nulliparous, 1, 2, 3, and 4 or more full-term pregnancies), ever use of oral contraceptives (no or yes), BMI at 1 year before diagnosis, and smoking status (never, former smokers that quit smoking for more than 1 year, and current smokers). Analyses were also adjusted on the first two principal components of the PCA. Because the results with and without principal components were similar, we only presented the results without adjustment on the principal components.

The p-values at the SNP and gene levels were corrected to take into account multiple testing using the false discovery rate (FDR) method (Benjamini and Hochberg, 1995). Statistical analyses were performed using R software.

## Results

### Characteristics of cases and controls

The sociodemographic characteristics of the cases and controls are presented in Table 1. As expected, the distribution across the matching variables (age and department of residence) was similar in cases and controls. The odds ratios for divorced/separated and widowed women were below 1. Years of education did not differ between cases and controls.

**TABLE 1:** Sociodemographic characteristics of cases and controls from the CATHY and YOUNG-THYR studies included in the analysis of genetic risk factors.

	Controls (n = 482)		Cases (n = 463)		OR^a^	95% CI
	n	%	n	%		
Age (years)						
<30	75	15.60%	59	12.70%		
30–34	68	14.10%	58	12.50%		
35–39	42	8.70%	39	8.40%		
40–44	50	10.40%	32	6.90%		
45–49	46	9.50%	54	11.70%		
50–54	54	11.20%	72	15.60%		
55–59	48	10.00%	55	11.90%		
60–64	31	6.40%	41	8.90%		
65–69	33	6.80%	24	5.20%		
70–74	20	4.10%	17	3.70%		
≥75	15	3.10%	12	2.60%		
Department of residence						
Ardennes	69	14.30%	83	17.90%		
Calvados	137	28.40%	124	26.80%		
Marne	183	38.00%	171	36.90%		
Other departments	93	19.30%	85	18.40%		
Family situation						
In couple or married	350	72.60%	356	76.90%	1	Reference
Single	47	9.80%	43	9.30%	1.01	[0.63, 1.62]
Divorced or separated	44	9.10%	39	8.40%	0.80	[0.50, 1.27]
Widow	41	8.50%	25	5.40%	**0.50**	**[0.28, 0.91]**
Years of education						
≤5	96	19.90%	102	22.00%	1	Reference
6–9	143	29.70%	162	35.00%	1.12	[0.76, 1.65]
10–12	88	18.30%	59	12.70%	0.68	[0.42, 1.09]
>12	155	32.20%	140	30.20%	0.96	[0.62, 1.46]

^a^ Odds ratios adjusted for age and department of residence.

OR, odds ratio; CI, confidence interval. Bold values indicate statistically significant odds ratios (95% CI not including 1).

Table 2 shows the odds ratios of DTC risk associated with selected risk factors. Increased ORs, although not statistically significant, were observed for family history of thyroid cancer in first-degree relatives, high BMI (≥25 kg/m^2^) 1 year before diagnosis, late age at menarche (≥15 years), and high parity (≥4 children). A reduced OR at the limit of statistical significance was observed for oral contraceptive use.

**TABLE 2:** Odds ratio of association between lifestyle risk factors and differentiated thyroid cancer risk.

	Controls (n = 482)	Cases (n = 463)	OR^a^	95% CI	OR^b^	95% CI
Family history of thyroid cancer						
No	473	449	1	Reference	1	Reference
Yes	9	14	1.82	[0.77, 4.32]	1.88	[0.77, 4.62]
BMI (kg/m^2^)						
<18.5	21	17	0.95	[0.48, 1.86]	0.89	[0.45, 1.78]
18.5–24.99	272	229	1	Reference	1	Reference
25–29.99	116	130	1.27	[0.93, 1.74]	1.29	[0.92, 1.80]
≥30	70	85	1.35	[0.93, 1.96]	1.37	[0.92, 2.02]
Missing values	3	2	-	-	-	-
Smoking status						
Never smoker	266	253	1	Reference	1	Reference
Former smoker	100	111	1.13	[0.81, 1.57]	1.17	[0.83, 1.66]
Current smoker	115	98	0.91	[0.65, 1.28]	0.98	[0.68, 1.41]
Missing values	1	1	-	-	-	-
Age at menarche (years)						
≤12	211	198	1	Reference	1	Reference
13	120	108	0.99	[0.71, 1.38]	1.05	[0.74, 1.48]
14	87	87	1.10	[0.77, 1.59]	1.11	[0.76, 1.62]
≥15	62	68	1.23	[0.82, 1.84]	1.35	[0.88, 2.06]
Missing values	2	2	-	-	-	-
Parity						
0	96	78	1	Reference	1	Reference
1	95	90	1.14	[0.72, 1.80]	1.2	[0.71, 2.00]
2	155	156	1.21	[0.77, 1.89]	1.27	[0.76, 2.11]
3	97	87	1.09	[0.66, 1.80]	1.14	[0.65, 2.02]
≥4	39	52	1.70	[0.93, 3.12]	1.86	[0.95, 3.63]
Ever used oral contraceptive						
No	97	113	1	Reference	1	Reference
Yes	384	345	0.68	[0.47, 0.99]	0.67	[0.45, 1.00]
Missing values	1	5	-	-	-	-

^a^ Odds ratios adjusted for age and department of residence.

^b^ Odds ratios adjusted for age, department of residence, years of education, marital status, family history of thyroid cancer, BMI, smoking status, age at first menarche, parity, and use of oral contraception.

OR, odds ratio; CI, confidence interval.

### Circadian genes

#### Pathway- and gene-level analysis

The p-values calculated by the ARTP method on the basis of the SNPs located in each circadian gene showed no significant association between DTC and any of the genes, both before and after FDR correction for the number of genes. The ARTP p-value calculated for the circadian pathway as a whole was also not significant (p = 0.97) (Table 3).

**TABLE 3:** p-value of the association between the circadian rhythm pathway and differentiated thyroid cancer at the gene and pathway levels.

			463 cases/482 controls	
Gene	Region	Number of SNPs^a^	p-value^b^	FDR p-value^c^
*ARNTL*	11p15	23	0.83	0.99
*BHLHE40*	3p26	8	0.34	0.99
*BHLHE41*	12p12	3	0.41	0.99
*BTRC*	10q24	13	0.39	0.99
*CLOCK*	4q12	11	0.81	0.99
*CRY1*	12q23	7	0.86	0.99
*CRY2*	11p11	9	0.46	0.99
*CSNK1D*	17q25	3	0.63	0.99
*CSNK1E*	22q13	10	0.95	0.99
*CUL1*	7q36	22	0.12	0.99
*FBXL3*	13q22	8	0.18	0.99
*FBXW11*	5q35	8	0.80	0.99
*NPAS2*	2q11	61	0.59	0.99
*NR1D1*	17q21	7	0.75	0.99
*PER1*	17p13	5	0.62	0.99
*PER2*	2q37	11	0.69	0.99
*PER3*	1p36	14	0.29	0.99
*RBX1*	22q13	2	0.99	0.99
*RORA*	15q22	285	0.41	0.99
*RORB*	9q21	35	0.96	0.99
*RORC*	1q21	13	0.27	0.99
*SKP1*	5q31	4	0.95	0.99
*TIMELESS*	12q13	8	0.97	0.99
Circadian Pathway		570 (23 genes)	0.97	

All analyses were adjusted for age, department of residence, years of education, marital status, BMI, smoking status, age at menarche, parity, and use of oral contraception.

^a^ Number of SNPs genotyped in the gene region (gene coordinates +/− 50 kb).

^b^ p-values calculated by the ARTP method.

^c^ p-values corrected by the FDR method.

#### SNP-level analysis

The p-values of association between DTC and each of the 570 SNPs located in the 23 circadian genes are shown in Supplementary Table S1. No SNP had a statistically significant p-value at the 0.05 threshold after FDR correction for the number of SNPs. The top SNPs were mostly located in *RORA*.

### Gene–environment interactions (GxE)

#### Pathway- and gene-level analysis

The p-values of the interaction between circadian genes and DTC risk factors are shown in Table 4. At the whole pathway level, a significant interaction with smoking status was detected (p = 0.04). At the gene level, an interaction with smoking status at the limit of statistical significance after FDR correction was observed for *PER3* (p = 0.05). No interaction between genes and other DTC risk factors examined was detected.

**TABLE 4:** p-value of interaction between the circadian rhythm pathway and suspected risk factors for DTC at the gene level and pathway levels.

Gene	Number of SNPs^a^	BMI		Parity		Age at menarche		Birth control pills		Smoking status	
		p-value^b^	FDR^c^	p-value^b^	FDR^c^	p-value^b^	FDR^c^	p-value^b^	FDR^c^	p-value^b^	FDR^c^
*ARNTL*	23	0.77	0.89	0.45	0.94	0.30	0.85	0.81	0.96	0.56	0.86
*BHLHE40*	8	0.85	0.89	0.38	0.87	0.15	0.69	0.18	0.70	0.91	0.91
*BHLHE41*	3	0.26	0.89	0.94	0.98	0.78	0.94	0.28	0.70	0.18	0.69
*BTRC*	13	0.46	0.89	0.98	0.98	0.89	0.94	0.21	0.70	0.57	0.86
*CLOCK*	11	0.89	0.89	0.81	0.98	0.39	0.94	0.14	0.70	0.64	0.86
*CRY1*	7	0.76	0.89	0.78	0.98	0.21	0.69	0.83	0.96	0.71	0.86
*CRY2*	9	0.67	0.89	0.95	0.98	0.54	0.94	0.81	0.96	0.18	0.69
*CSNK1D*	3	0.05	0.53	0.72	0.98	0.73	0.94	0.40	0.70	0.85	0.90
*CSNK1E*	10	0.83	0.89	0.76	0.98	0.21	0.69	0.88	0.96	0.40	0.77
*CUL1*	22	0.65	0.89	0.37	0.87	0.63	0.94	0.09	0.70	0.03	0.24
*FBXL3*	8	0.46	0.89	0.94	0.98	0.90	0.94	0.47	0.72	0.60	0.86
*FBXW11*	8	0.15	0.67	0.06	0.48	0.74	0.94	0.26	0.70	0.69	0.86
*NPAS2*	61	0.80	0.89	0.90	0.98	0.79	0.94	0.32	0.70	0.86	0.90
*NR1D1*	7	0.44	0.89	0.22	0.87	0.09	0.69	0.96	0.96	0.41	0.77
*PER1*	5	0.51	0.89	0.13	0.75	0.60	0.94	0.54	0.73	0.26	0.75
*PER2*	11	0.02	0.44	0.04	0.48	0.75	0.94	0.20	0.70	0.83	0.90
*PER3*	14	0.11	0.64	0.73	0.98	1.00	1.00	0.96	0.96	**0.002**	**0.05**
*RBX1*	2	0.07	0.53	0.26	0.87	0.51	0.94	0.34	0.70	0.37	0.77
*RORA*	285	0.60	0.89	0.30	0.87	0.51	0.94	0.47	0.72	0.44	0.77
*RORB*	35	0.64	0.89	0.95	0.98	0.13	0.69	0.51	0.73	0.26	0.75
*RORC*	13	0.47	0.89	0.05	0.48	0.11	0.69	0.04	0.70	**0.01**	**0.09**
*SKP1*	4	0.40	0.89	0.36	0.87	0.87	0.94	0.25	0.70	0.11	0.64
*TIMELESS*	8	0.83	0.89	0.62	0.98	0.10	0.69	0.37	0.70	0.40	0.77
Circadian pathway	570 (23 genes)	0.41		0.53		0.70		0.64		**0.04**	

All analyses are adjusted for age, department of residence, BMI, smoking status, Age at menarche, parity, and use of oral contraception.

^a^ Number of SNPs genotyped in the gene region (gene coordinates +/− 50 kb).

^b^ p-values calculated by the ARTP method.

^c^ p-values corrected by the false discovery rate (FDR) method (correction for the 23 genes tested). Bold p-values indicate statistically significant interaction after false discovery rate (FDR) correction.

#### SNP-level analysis

We also tested the interaction between smoking status and circadian polymorphisms at the SNP level (Supplementary Table S2). Of the 570 SNPs, the interaction p-value with smoking status was significant after FDR correction for rs11204897 in *RORC* (p = 0.04) and two SNPs, rs1012477 and rs10462018, located in *PER3* (p = 0.04 for both). These two SNPs are in linkage disequilibrium (LD) (r^2^ = 1); therefore, only results for rs1012477 are shown.

We finally calculated the ORs of the association between these SNPs and DTC separately in current smokers, ex-smokers, and non-smokers (Table 5). For rs1012477 in *PER3*, the OR comparing the mutated allele to the wild-type allele was increased in ex-smokers and decreased in current smokers. For the SNP in *RORC*, the OR was significantly increased in non-smokers and ex-smokers, and it was significantly decreased in current smokers.

**TABLE 5:** Odds ratios of association between rs1012477, rs10462018, rs11204897, and DTC according to the smoking status.

							Total (463 C/482 T)	Never-smokers (253 C/266 T)	Former smokers (111 C/100 T)	Current smokers (98 C/115 T)
CHROM	POS	Gene	SNP	EA	OA	EAF	OR^a^ [IC 95%]	OR^a^ [IC 95%]	OR^a^ [IC 95%]	OR^a^ [IC 95%]
Uni-locus model										
1	7,858,135	*PER3*	rs1012477	G	C	0.13	0.80 [0.60; 1.05]	0.74 [0.50; 1.09]	**2.79 [1.34, 5.80]**	**0.38 [0.20, 0.73]**
7	151,809,066	*RORC*	rs11204897	G	A	0.14	1.23 [0.93; 1.63]	**1.48 [1.01, 2.17]**	**2.25 [1.17, 4.34]**	**0.31 [0.14, 0.67]**
Multi-loci model										
1	7,858,135	PER3	rs1012477	G	C	0.13	0.79 [0.60; 1.04]	0.73 [0.49, 1.08]	**2.92 [1.39, 6.14]**	**0.41 [0.21, 0.79]**
7	151,809,066	RORC	rs11204897	G	A	0.14	1.25 [0.94; 1.65]	**1.49 [1.02, 2.19]**	**2.37 [1.21, 4.65]**	**0.33 [0.15, 0.73]**

^a^ Odds ratios adjusted for age, department of residence, BMI, age at menarche, parity, and use of oral contraception.

CHROM, chromosome; POS, SNP position (genome build 37); EA, effect allele; OA, other allele; EAF, effect allele frequency; OR, odds ratio; CI, confidence interval. Bold odds ratios highlight statistically significant associations (95% CI not including 1).

## Discussion

The objective of our study was to explore the risk of DTC in relation to circadian gene polymorphisms and the interactions between circadian genes and DTC risk factors. Overall, we found no association between circadian gene variants and DTC risk at the SNP, gene, and pathway levels. However, we found that smoking status may modulate the risk of DTC associated with circadian genes. More specifically, the interaction between smoking status and genes detected for the whole circadian pathway was mostly driven by rs1012477 and rs10462018 in *PER3* and rs11204897 in *RORC*. To the best of our knowledge, this is the first study that specifically examined the role of circadian gene polymorphisms in DTC risk.

Circadian genes are key regulators of the circadian system, which is generated through transcription–translation feedback loops initiated by the ARTNL–CLOCK and ARNTL–NPAS2 heterodimers; these transcriptional factors induce the expression of *PERs*, *CRYs*, *NR1Ds*, and *RORs*. *PER*s and *CRYs* will act on the ARNTL–CLOCK/NPAS2 complex to repress their own transcription. On a secondary loop, RORA activates the transcription of *ARNTL*, whereas NR1D1 and NR1D2 repress it. In addition, the ARNTL–CLOCK/NPAS2 complex also regulates the transcription of several downstream clock-controlled genes that are involved in various biological functions, including some that are relevant to carcinogenesis, such as cell-cycle control or DNA damage repair.

In previous studies, several circadian gene polymorphisms have been reported to be associated with different types of cancer, such as breast, prostate, ovarian, pancreatic, or colorectal cancer (Morales-Santana et al., 2019). The associations between circadian genes and breast or prostate cancer have been examined most frequently. Polymorphisms in *CLOCK* (Hoffman et al., 2010; Truong et al., 2014), *NPAS2* (Zhu et al., 2008), *CRY2* (Hoffman et al., 2010), *RORA* (Truong et al., 2014), *TIMELESS* (Fu et al., 2012), and *ARNTL* (Zienolddiny et al., 2013) have been associated with breast cancer. Studies using the gene set analysis method confirmed an association with *ARNTL*, *CLOCK*, and *RORA* and additionally reported an association with *RORB*, *RORC*, *CRY*, and the overall pathway in relation to breast cancer risk (Truong et al., 2014; Mocellin et al., 2018). Only two previous studies carried out a comprehensive analysis on the association between circadian gene polymorphisms and prostate cancer risk using the same definition of the pathway that we used. These two studies reported a significant association at the gene level for *ARNTL*, *NPAS2*, *RORA*, and *PER1* (Zhu et al., 2009; Wendeu-Foyet et al., 2019), as well as for the whole pathway (Mocellin et al., 2018; Wendeu-Foyet et al., 2019).

Studies on breast and prostate cancer on the interaction between night-shift work and circadian genes (Monsees et al., 2012; Grundy et al., 2013; Zienolddiny et al., 2013; Rabstein et al., 2014; Truong et al., 2014; Wendeu-Foyet et al., 2020) reported that polymorphisms in circadian genes *ARNTL*, *CLOCK*, *CRY2*, *CSNK1E*, *NPAS2*, *PER2*, *PER3*, *RORA*, and *RORB* were associated with the disease, particularly in night workers, i.e., individuals who are more subject to the disruption of circadian rhythms. The circadian clock is regulated at the molecular level by circadian genes, but external factors such as light exposure or lifestyle factors such as timing of food intake, sleep patterns, and work schedules also contribute to regulating the circadian rhythms.

In our study, we reported a significant interaction between smoking status and SNPs in *PER3* (rs1012477 and rs10462018, r^2^= 1) and *RORC* (rs11204897). Smoking is usually inversely associated with thyroid cancer risk in observational studies, and the underlying biological mechanism is poorly understood (Kitahara and Schneider, 2022). In our study, the OR for current smokers was below 1, but the association was not significant. In a previous study based on the UK Biobank data (Gibson et al., 2019), the authors reported a moderate genetic correlation between smoking and sleep behaviors (sleep duration, chronotype, and insomnia), and using Mendelian randomization analyses, they found that heavier smoking causally decreases the likelihood of being a morning person, whereas insomnia increases smoking intensity, suggesting a bidirectional link between smoking and sleep behaviors. It is, therefore, possible that smoking status reflects some disruption of the circadian rhythms in our study.

It is also interesting to note that rs1012477 (located in an intron of *PER3*) was previously found to be associated with breast cancer (Zienolddiny et al., 2013) and prostate cancer risk (Zhu et al., 2009). Other polymorphisms located in *PER3* were associated with breast, prostate, and colorectal cancers (Zhu et al., 2009; Zienolddiny et al., 2013; Alexander et al., 2015) and sleep disorders (Archer et al., 2018). Studies suggested that the expression of *PER3* may be altered in certain cancers (Wang et al., 2012; Liu et al., 2014; Fores-Martos et al., 2021); however, the precise role of *PER3* in cancer development is not known.

The RORC protein is one of the family members of the nuclear orphan receptors that function as transcription factors. Expression of RORC was found to be significantly decreased in several types of cancer cells, indicating a probable regulatory role of RORC in tumorigenesis (Cao et al., 2019).

The strengths of our study include a comprehensive selection of SNPs and genes in the circadian pathway. We selected 570 variants from 23 genes in this pathway, which ensured a high coverage of the genetic variations in this pathway, whereas most previous studies focused on approximately 10 circadian genes.

We also used a pathway approach to analyze the overall effect of circadian genes on thyroid cancer risk. This approach is useful for detecting the combined effects of genetic polymorphisms that are individually weakly associated with disease and can provide additional hypotheses about the underlying mechanisms. In addition, as circadian rhythms are generated by multiple molecular interactions, SNP analyses may not be sufficient to analyze the genetic complexity of the association between circadian genes and DTC cancer.

Our study was based on datasets with detailed epidemiological information, but it also has some limitations. A relatively small study sample limited the statistical power of the analyses. Because our study was based on a subsample of participants in the CATHY and YOUNG-THYR studies, we have checked that the characteristics of the genotyped population were almost identical to those of the total population from which it was derived (Supplementary Tables S3, S4). Only the association with oral contraceptive use differs between the overall sample and the genotyped sample, but all analyses were adjusted for this variable in addition to other confounding variables associated with DTC risk. However, some important variables relative to circadian disruption, such as sleep behavior and night work, were not available in the current study, but, to the best of our knowledge, this is the only study that specifically analyzed the circadian gene polymorphisms and their interaction with non-genetic factors in DTC risk.

Although our study focused on circadian gene polymorphisms, it is important to consider these findings in the broader context of known genetic alterations in differentiated thyroid cancer. Somatic mutations such as *BRAF* V600E, *TERT* promoter mutations, and gene fusions involving *RET* or *NTRK* play crucial roles in DTC pathogenesis and influence its clinical behavior (Mady et al., 2020; Truong and Lesueur, 2022; Nannini et al., 2023). It would be interesting to explore potential interactions between circadian gene polymorphisms and these somatic alterations. For instance, circadian rhythm disruptions might modulate the expression or activity of oncogenes such as *BRAF* or *RET*, or they might influence DNA repair mechanisms involved in acquiring secondary mutations. Smoking status, which we identified as a modulator of the association between circadian polymorphisms and DTC, might also interact with these somatic alterations. Future studies integrating both germline variations in circadian genes and somatic mutation profiles could provide valuable insights into the molecular mechanisms underlying thyroid carcinogenesis and potentially identify new therapeutic targets or prognostic markers.

## Conclusion

This study suggests that smoking status may modulate the association between DTC and polymorphisms in some of the circadian genes (*PER3* and *RORC*). If this association is confirmed, new biological hypotheses on the biological function of circadian genes will have to be explored as the underlying mechanisms are currently unknown.

## Data Availability

The original contributions presented in the study are included in the article/Supplementary Material; further inquiries can be directed to the corresponding author.
