# Supplementary material for: Polymorphisms in circadian rhythm genes and the risk of differentiated thyroid cancer
Source: Front Genet. 2025 Jun 4;16:1539090. doi: 10.3389/fgene.2025.1539090 (PMC12174421; doi:10.3389/fgene.2025.1539090)
Supplement: Supplementary file 1 [file Table1.docx]

**Supplementary table 1: Odds Ratio and P-value of association of the most significant SNPs in circadian pathway with thyroid cancer risk.**^a^: Odds ratios are adjusted for age, department of residence, BMI, smoking status, age at menarche, number of full-term pregnancies, and use of oral contraception. ^b^: p-values corrected by the false discovery rate (correction for the 570 SNPs tested). CHROM: chromosome; POS: SNP position (genome build 37); EA: effect allele, OA: Other allele; EAF: effect allele frequency; OR: Odds-ratio; CI: Confidence interval.

| **CHROM** | **POS** | **SNP** | **Location** | **Gene** | **EA** | **OA** | **EAF** | **BETA** | **SE** | **OR^a^** | **IC95** | **P** | **FDR (BH)^b^** |
| --- | --- | --- | --- | --- | --- | --- | --- | --- | --- | --- | --- | --- | --- |
| 15 | 61028486 | rs11635314 | INTRON | RORA | A | G | 0,15 | 0,48 | 0,14 | 1,62 | [ 1.243 ; 2.114 ] | 3,70E-04 | 0,211 |
| 15 | 61029620 | rs341413 | INTRON | RORA | G | A | 0,20 | 0,34 | 0,12 | 1,41 | [ 1.118 ; 1.783 ] | 3,82E-03 | 0,994 |
| 7 | 148395124 | rs243551 | COMPLEX | CUL1 | G | C | 0,41 | 0,27 | 0,10 | 1,31 | [ 1.073 ; 1.594 ] | 7,83E-03 | 0,994 |
| 15 | 61240545 | rs4332688 | INTRON | RORA | C | G | 0,52 | -0,25 | 0,10 | 0,78 | [ 0.646 ; 0.945 ] | 1,11E-02 | 0,994 |
| 15 | 61209011 | rs8036866 | INTRON | RORA | A | G | 0,10 | -0,41 | 0,17 | 0,67 | [ 0.481 ; 0.923 ] | 1,47E-02 | 0,994 |
| 15 | 60862962 | rs8041381 | INTRON | RORA | G | A | 0,35 | 0,23 | 0,10 | 1,27 | [ 1.037 ; 1.543 ] | 2,06E-02 | 0,994 |
| 15 | 60990883 | rs12438414 | INTRON | RORA | A | G | 0,22 | 0,27 | 0,12 | 1,31 | [ 1.041 ; 1.655 ] | 2,13E-02 | 0,994 |
| 15 | 61263377 | rs17204698 | INTRON | RORA | C | A | 0,20 | 0,28 | 0,12 | 1,32 | [ 1.041 ; 1.685 ] | 2,23E-02 | 0,994 |
| 15 | 61060707 | rs12591650 | INTRON | RORA | G | A | 0,16 | 0,30 | 0,13 | 1,35 | [ 1.043 ; 1.757 ] | 2,26E-02 | 0,994 |
| 15 | 61248951 | rs2140441 | INTRON | RORA | T | A | 0,39 | 0,23 | 0,10 | 1,26 | [ 1.031 ; 1.528 ] | 2,37E-02 | 0,994 |
| 13 | 77594330 | rs636646 | INTRON | FBXL3 | G | C | 0,24 | 0,26 | 0,12 | 1,30 | [ 1.034 ; 1.627 ] | 2,43E-02 | 0,994 |
| 1 | 7884580 | rs697690 | INTRON | PER3 | G | A | 0,32 | -0,23 | 0,10 | 0,79 | [ 0.649 ; 0.973 ] | 2,59E-02 | 0,994 |
| 2 | 101522266 | rs4851377 | INTRON | NPAS2 | G | A | 0,47 | -0,21 | 0,10 | 0,81 | [ 0.670 ; 0.976 ] | 2,69E-02 | 0,994 |
| 15 | 60943212 | rs339996 | INTRON | RORA | G | A | 0,45 | 0,21 | 0,10 | 1,24 | [ 1.023 ; 1.496 ] | 2,85E-02 | 0,994 |
| 1 | 151783688 | rs939595 | INTRON | RORC | A | C | 0,38 | -0,21 | 0,10 | 0,81 | [ 0.670 ; 0.984 ] | 3,33E-02 | 0,994 |
| 15 | 60843771 | rs4775281 | INTRON | RORA | A | C | 0,39 | -0,22 | 0,10 | 0,81 | [ 0.662 ; 0.983 ] | 3,33E-02 | 0,994 |
| 15 | 61057225 | rs2279295 | INTRON | RORA | G | A | 0,43 | -0,20 | 0,10 | 0,82 | [ 0.675 ; 0.985 ] | 3,42E-02 | 0,994 |
| 2 | 101617493 | rs10189697 | INTERGENIC | NPAS2 | G | C | 0,24 | 0,23 | 0,11 | 1,26 | [ 1.012 ; 1.575 ] | 3,87E-02 | 0,994 |
| 15 | 61399892 | rs782920 | INTRON | RORA | A | G | 0,06 | -0,45 | 0,22 | 0,64 | [ 0.415 ; 0.984 ] | 4,22E-02 | 0,994 |
| 10 | 103319198 | rs11191048 | INTERGENIC | BTRC | A | G | 0,19 | 0,24 | 0,12 | 1,27 | [ 1.006 ; 1.612 ] | 4,48E-02 | 0,994 |
| 3 | 5030837 | rs2137946 | INTERGENIC | BHLHE40 | A | G | 0,23 | -0,23 | 0,12 | 0,79 | [ 0.634 ; 0.995 ] | 4,54E-02 | 0,994 |
| 15 | 61062967 | rs12914272 | INTRON | RORA | A | G | 0,40 | 0,20 | 0,10 | 1,22 | [ 1.003 ; 1.477 ] | 4,71E-02 | 0,994 |

**Supplementary table 2: P-values of the most significant SNPs x smoking status (Non-smoker, Former smoker, Current smoker).**

^a^:P.int: p-value; ^b^: p-values corrected by the false discovery rate (correction for the 570 SNPs tested). CHROM: chromosome; POS: SNP position (genome build 37); EA: effect allele, OA: Other allele; EAF: effet allele frequency; OR: Odds-ratio; CI: Confidence interval.

| **CHROM** | **POS** | **SNP** | **Location** | **Gene** | **EA** | **OA** | **EAF** | **Z** | **P.int^a^** | **FDR (BH)^b^** |
| --- | --- | --- | --- | --- | --- | --- | --- | --- | --- | --- |
| 1 | 151809066 | rs11204897 | INTERGENIC | RORC | G | A | 0,14 | 16,58 | 2,51E-04 | 0,049 |
| 1 | 7858135 | rs1012477 | INTRON | PER3 | G | C | 0,13 | 16,53 | 2,57E-04 | 0,049 |
| 1 | 7879627 | rs10462018 | INTRON | PER3 | A | G | 0,13 | 16,53 | 2,57E-04 | 0,049 |
| 7 | 148499341 | rs12154650 | INTERGENIC | CUL1 | G | C | 0,02 | 14,62 | 6,69E-04 | 0,095 |
| 15 | 61208729 | rs6494232 | INTRON | RORA | G | A | 0,08 | 12,40 | 2,03E-03 | 0,231 |
| 15 | 61480721 | rs17204952 | INTRON | RORA | A | G | 0,18 | 10,86 | 4,39E-03 | 0,417 |
| 1 | 7870048 | rs228669 | CODING | PER3 | A | G | 0,08 | 10,42 | 5,45E-03 | 0,431 |
| 15 | 60983676 | rs880625 | INTRON | RORA | G | A | 0,14 | 10,22 | 6,04E-03 | 0,431 |
| 15 | 60794649 | rs12594972 | INTRON | RORA | G | A | 0,09 | 09,45 | 8,86E-03 | 0,532 |
| 15 | 60799909 | rs17270188 | INTRON | RORA | G | A | 0,37 | 09,09 | 1,06E-02 | 0,532 |
| 1 | 7879168 | rs228689 | INTRON | PER3 | A | G | 0,11 | 08,99 | 1,12E-02 | 0,532 |
| 9 | 77283889 | rs1327837 | INTRON | RORB | A | G | 0,35 | 08,86 | 1,19E-02 | 0,532 |
| 1 | 7840926 | rs697679 | UTR | PER3 | C | G | 0,24 | 08,70 | 1,29E-02 | 0,532 |
| 1 | 7841549 | rs697680 | INTERGENIC | PER3 | A | C | 0,15 | 08,48 | 1,44E-02 | 0,532 |
| 15 | 61506685 | rs930358 | INTRON | RORA | G | A | 0,20 | 08,41 | 1,49E-02 | 0,532 |
| 15 | 60977195 | rs17303097 | INTRON | RORA | C | A | 0,19 | 08,37 | 1,52E-02 | 0,532 |
| 5 | 133514972 | rs3776841 | INTERGENIC | SKP1 | C | A | 0,09 | 08,29 | 1,59E-02 | 0,532 |
| 15 | 60954024 | rs972531 | INTRON | RORA | G | A | 0,40 | 07,07 | 2,91E-02 | 0,823 |
| 9 | 77263592 | rs17060387 | INTRON | RORB | A | G | 0,15 | 06,94 | 3,12E-02 | 0,823 |
| 15 | 61248951 | rs2140441 | INTRON | RORA | T | A | 0,39 | 06,91 | 3,16E-02 | 0,823 |
| 11 | 45906830 | rs1554338 | INTRON | CRY2 | G | A | 0,06 | 06,87 | 3,23E-02 | 0,823 |
| 15 | 61269447 | rs8031801 | INTRON | RORA | A | G | 0,23 | 06,78 | 3,37E-02 | 0,823 |
| 15 | 61452749 | rs1437541 | INTRON | RORA | A | G | 0,09 | 06,69 | 3,52E-02 | 0,823 |
| 15 | 60792942 | rs7183916 | INTRON | RORA | G | A | 0,19 | 06,68 | 3,54E-02 | 0,823 |
| 9 | 77282927 | rs1327838 | INTRON | RORB | G | A | 0,20 | 06,64 | 3,61E-02 | 0,823 |
| 15 | 61312022 | rs17303334 | INTRON | RORA | A | C | 0,15 | 06,53 | 3,82E-02 | 0,836 |
| 15 | 61495982 | rs726955 | INTRON | RORA | A | G | 0,26 | 06,45 | 3,97E-02 | 0,838 |
| 17 | 8041704 | rs9303226 | INTERGENIC | PER1 | C | G | 0,42 | 06,37 | 4,13E-02 | 0,842 |
| 15 | 61373581 | rs782905 | INTRON | RORA | A | T | 0,22 | 06,20 | 4,50E-02 | 0,876 |
| 15 | 61472606 | rs1370430 | INTRON | RORA | A | G | 0,37 | 06,04 | 4,88E-02 | 0,876 |
| 1 | 151792984 | rs11578418 | INTRON | RORC | A | G | 0,09 | 06,00 | 4,97E-02 | 0,876 |

**Supplementary table 3: Odds ratio of association between lifestyle risk factors and thyroid cancer risk in the total population and the genotyped population (CATHY study).**
OR: Odds-ratio; CI: Confidence interval

|  | **988 women (Total)** | | | | **785 women (Genotyped)** | | | |
| --- | --- | --- | --- | --- | --- | --- | --- | --- |
|  | **Controls  (n=505)** | **Cases  (n=483)** | **OR*** | **95% CI** | **Controls  (n=391)** | **Cases  (n=394)** | **OR*** | **95% CI** |
| **Family situation** |  |  |  |  |  |  |  |  |
| In couple or Married | 363 | 365 | 1,00 | Reference | 290 | 312 | 1,00 | Reference |
| Single | 30 | 29 | 1,13 | [0.65 - 1.97] | 21 | 20 | 1,00 | [0.52 - 1.94] |
| Divorced or Separated | 54 | 51 | 0,86 | [0.56 - 1.31] | 40 | 37 | 0,78 | [0.48 - 1.27] |
| Widow | 58 | 38 | 0,54 | [0.33 - 0.87] | 40 | 25 | 0,51 | [0.28 - 0.92] |
| **Years of education** |  |  |  |  |  |  |  |  |
| ≤5 | 130 | 136 | 1,00 | Reference | 95 | 105 | 1,00 | Reference |
| 6-9 | 166 | 180 | 1,10 | [0.77 - 1.55] | 132 | 145 | 1,03 | [0.70 - 1.53] |
| 10-12 | 81 | 53 | 0,68 | [0.43 - 1.07] | 66 | 48 | 0,72 | [0.44 - 1.184] |
| >12 | 126 | 114 | 0,99 | [0.66 - 1.48] | 98 | 96 | 1,03 | [0.66 - 1.62] |
| missing value | 2 | 0 | - | - | 0 | 0 | - | - |
| **Smoking status** |  |  |  |  |  |  |  |  |
| Non-smoker | 297 | 284 | 1,00 | Reference | 227 | 223 | 1,00 | Reference |
| Former smoker | 106 | 118 | 1,13 | [0.82 - 1.55] | 83 | 101 | 1,19 | [0.83 - 1.70] |
| Current smoker | 102 | 80 | 0,82 | [0.58 - 1.18] | 81 | 69 | 0,87 | [0.58 - 1.29] |
| missing value | 0 | 1 | - | - | 0 | 1 | - | - |
| **Family history of thyroid cancer** |  |  |  |  |  |  |  |  |
| No | 496 | 465 | 1,00 | Reference | 384 | 381 | 1,00 | Reference |
| Yes | 9 | 18 | 2,52 | [1.10 - 5.78] | 7 | 13 | 2,17 | [0.84 - 5.62] |
| **Age at menarche** |  |  |  |  |  |  |  |  |
| ≤12 | 213 | 189 | 1,00 | Reference | 168 | 160 | 1,00 | Reference |
| 13 | 125 | 117 | 1,06 | [0.77 - 1.47] | 95 | 91 | 1,05 | [0.73 - 1.52] |
| 14 | 95 | 93 | 1,13 | [0.79 - 1.61] | 73 | 78 | 1,19 | [0.80 - 1.77] |
| ≥15 | 69 | 82 | 1,38 | [0.94 - 2.02] | 53 | 63 | 1,33 | [0.86 - 2.06] |
| missing value | 3 | 2 | - | - | 2 | 2 | - | - |
| **Number of full-term pregnancies** |  |  |  |  |  |  |  |  |
| 0 | 53 | 44 | 1,00 | Reference | 42 | 32 | 1,00 | Reference |
| 1 | 90 | 93 | 1,13 | [0.68 - 1.89] | 71 | 76 | 1,33 | [0.74 - 2.36] |
| 2 | 186 | 171 | 1,00 | [0.62 - 1.62] | 141 | 144 | 1,24 | [0.72 - 2.14] |
| 3 | 121 | 107 | 0,96 | [0.57 - 1.60] | 97 | 88 | 1,11 | [0.62 - 1.97] |
| ≥4 | 55 | 68 | 1,38 | [0.77 - 2.46] | 40 | 54 | 1,75 | [0.90 - 3.39] |
| **Ever used oral contraceptive** |  |  |  |  |  |  |  |  |
| No | 119 | 138 | 1,00 | Reference | 90 | 104 | 1,00 | Reference |
| Yes | 384 | 340 | 0,68 | [0.48 - 0.97] | 300 | 285 | 0,71 | [0.47 - 1.05] |
| missing value | 2 | 5 | - | - | 1 | 5 | - | - |
| **BMI (Kg/m²)** |  |  |  |  |  |  |  |  |
| <18.5 | 19 | 14 | 0,93 | [0.45 - 1.91] | 12 | 10 | 0,95 | [0.40 - 2.28] |
| 18.5-24.99 | 271 | 230 | 1,00 | Reference | 208 | 182 | 1,00 | Reference |
| 25-29.99 | 129 | 144 | 1,25 | [0.92 - 1.70] | 105 | 119 | 1,23 | [0.87 - 1.72] |
| ≥30 | 82 | 92 | 1,22 | [0.86 - 1.75] | 63 | 80 | 1,35 | [0.57 - 3.54] |
| missing value | 4 | 3 | - | - | 3 | 3 | - | - |

**Supplementary table 4: Odds ratio of association between lifestyle risk factors and thyroid cancer risk in the total population and the genotyped population (YOUNG-THYR study).**OR: Odds-ratio; CI: Confidence interval

|  | **1312 women (Total)** | | | | **192 women (Genotyped)** | | | |
| --- | --- | --- | --- | --- | --- | --- | --- | --- |
|  | **Controls  (n= 679)** | **Case  (n= 633)** | **OR*** | **95% CI** | **Controls  (n= 97)** | **Case  (n= 95)** | **OR*** | **95% CI** |
|  |  |  |  |  |  |  |  |  |
| **Family situation** |  |  |  |  |  |  |  |  |
| In couple or Married | 454 | 407 | 1,00 | Reference | 64 | 61 | 1,00 | Reference |
| Single | 201 | 187 | 1,04 | [0.81 - 1.33] | 28 | 27 | 1,01 | [0.52 - 1.95] |
| Divorced or Separated | 21 | 36 | 1,91 | [1.10 - 3.33] | 4 | 7 | 1,85 | [0.51 - 6.70] |
| Widow | 3 | 2 | 0,75 | [0.12 - 4.54] | 1 | 0 | - | - |
| missing value | 0 | 1 |  |  |  |  |  |  |
| **Years of education** |  |  |  |  |  |  |  |  |
| ≤5 | 14 | 25 | 1,00 | Reference | 2 | 3 | 1,00 | Reference |
| 6-9 | 117 | 164 | 0,78 | [0.39 - 1.57] | 12 | 26 | 1,45 | [0.21 - 9.85] |
| 10-12 | 136 | 128 | 0,53 | [0.26 - 1.06] | 24 | 15 | 0,42 | [0.06 - 2.79] |
| >12 | 412 | 316 | 0,43 | [0.22 - 0.84] | 59 | 51 | 0,58 | [0.09 - 3.59] |
| **Smoking status** |  |  |  |  |  |  |  |  |
| Non-smoker | 325 | 327 | 1,00 | Reference | 44 | 45 | 1,00 | Reference |
| Former smoker | 94 | 101 | 1,07 | [0.77 - 1.48] | 17 | 17 | 0,98 | [0.44 - 2.17] |
| Current smoker | 259 | 203 | 0,78 | [0.61 - 0.99] | 35 | 33 | 0,92 | [0.49 - 1.74] |
| missing value | 1 | 2 | - | - | 1 | 0 | - | - |
| **Family history of thyroid cancer** |  |  |  |  |  |  |  |  |
| No | 657 | 601 | 1,00 | Reference | 94 | 93 | 1,00 | Reference |
| Yes | 22 | 32 | 1,59 | [0.91 - 2.77] | 3 | 2 | 0,67 | [0.11 - 4.13] |
| **Age at menarche** |  |  |  |  |  |  |  |  |
| ≤12 | 322 | 325 | 1,00 | Reference | 47 | 52 | 1,00 | Reference |
| 13 | 177 | 157 | 0,88 | [0.67 - 1.14] | 25 | 22 | 0,79 | [0.39 - 1.59] |
| 14 | 101 | 83 | 0,81 | [0.59 - 1.13] | 14 | 15 | 0,97 | [0.42 - 2.22] |
| ≥15 | 74 | 59 | 0,79 | [0.54 - 1.15] | 11 | 6 | 0,49 | [0.17 - 1.43] |
| missing value | 5 | 9 | - | - | - | - | - | - |
| **Number of full-term pregnancies** |  |  |  |  |  |  |  |  |
| 0 | 444 | 387 | 1,00 | Reference | 57 | 54 | 1,00 | Reference |
| 1 | 138 | 124 | 1,08 | [0.81 - 1.45] | 24 | 19 | 0,86 | [0.40 - 1.84] |
| 2 | 80 | 91 | 1,42 | [0.99 - 2.04] | 14 | 16 | 1,26 | [0.52 - 3.04] |
| 3 | 16 | 25 | 2,00 | [1.02 - 3.90] | 2 | 5 | 2,79 | [0.48 - 16.14] |
| ≥4 | 1 | 6 | 7,34 | [0.87 - 61.53] | 0 | 1 | - | - |
| **Ever used oral contraceptive** |  |  |  |  |  |  |  |  |
| No | 99 | 127 | 1,00 | Reference | 89 | 78 | 1,00 | Reference |
| Yes | 580 | 506 | 0,67 | [0.50 - 0.90] | 8 | 17 | 2,53 | [1.01 - 6.30] |
| **BMI (Kg/m²)** |  |  |  |  |  |  |  |  |
| <18.5 | 75 | 57 | 0,87 | [0.60 - 1.26] | 9 | 9 | 1,19 | [0.44 - 3.22] |
| 18.5-24.99 | 459 | 402 | 1,00 | Reference | 67 | 56 | 1,00 | Reference |
| 25-29.99 | 96 | 106 | 1,26 | [0.93 - 1.71] | 13 | 20 | 1,85 | [0.84 - 4.05] |
| ≥30 | 48 | 67 | 1,59 | [1.07 - 2.36] | 8 | 10 | 1,50 | [0.55 - 4.05] |
| missing value | 1 | 1 | - | - | - | - | - | - |
